# Mediation role of telomere length in the relationship between physical activity and PhenoAge: A population-based study

**DOI:** 10.1016/j.jesf.2025.03.004

**Published:** 2025-03-26

**Authors:** Yanwei You, Dizhi Wang, Hao Ding, Weizhao Wang, Qiyu Liu, Danyi Zhang, Yuquan Chen, Xindong Ma

**Affiliations:** a Division of Sports Science & Physical Education, Tsinghua University, Beijing, 100084, China; b IDG/McGovern Institute for Brain Research, Tsinghua University, Beijing, 100084, China; c Saw Swee Hock School of Public Health, National University of Singapore, Singapore 117549, Singapore; d Department of Physical Education, Guangxi University of Chinese Medicine, Guangxi, 530200, China; e School of Life Sciences, Tsinghua University, Beijing, 100084, China; f School of Medical and Health Engineering, Changzhou University, Changzhou, 213164, China; g Department of Epidemiology & Preventive Medicine Alfred Hospital, Faculty of Medicine, Nursing & Health Sciences, Monash University, Victoria, 3800, Australia

**Keywords:** Physical activity, Telomere length, Phenotypic age, Aging, Mediation analysis

## Abstract

**Background:**

The relationship between physical activity (PA), telomere length, and phenotypic age (PhenoAge) represents a pivotal area of investigation in aging research.

**Methods:**

The study encompassed a cohort of 6200 participants aged 20 years and above, sourced from the National Health and Nutrition Examination Survey (NHANES). Physical activity (PA) levels were assessed employing the Global Physical Activity Questionnaire, while DNA samples were collected to determine telomere length, measured in base pairs. PhenoAge, an emerging aging index relying on nine distinct chemical biomarkers, was computed.

**Results:**

Incorporating a fully adjusted model, our analysis showed significant correlations between PA engagement and PhenoAge [Low PA, β (95 % CI): 0.039(-0.071,-0.008), p = 0.021; Moderate PA, β (95 % CI): 0.058(-0.082,-0.034), p < 0.001; High PA, β (95 % CI): 0.069(-0.096,-0.042), p < 0.001]. Furthermore, a positive link emerged between elevated PA levels and telomere length, with a β (95 % CI) of 0.011(0.001, 0.022), p = 0.034. A mediation analysis was performed, demonstrating that telomere length mediated the connection between PA and PhenoAge, with a proportion mediated calculated at 3.57 %.

**Conclusions:**

Our findings suggest that PA may play a key role in mitigating aging processes by preserving telomere length, highlighting the potential of PA as a target for interventions aimed at promoting healthy aging and longevity.

## Highlights

• Large nationwide population-based study.
• PA levels are positively associated with telomere length.
• PA levels are negatively associated with PhenoAge.
• Telomere length mediated the relationship between PA and PhenoAge.

## Graphical abstract

**Figure:**
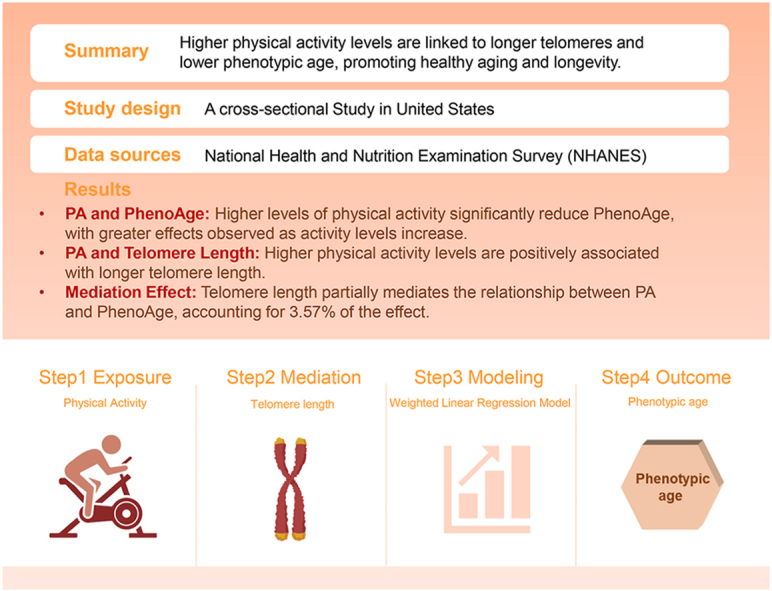

## 1 Introduction

The global demographic landscape is undergoing a shift towards an older population, driven by lengthening life expectancies and declining fertility rates. From an economic standpoint, a mere 2.2-year delay in the aging process could yield savings of up to seven trillion dollars over the ensuing half-century.^1^ Aging, as a phenomenon, involves a wide range of changes in body composition, internal balance, energy dynamics, and cerebral well-being.^2^ Empirical evidence underscores the potential for aging to contribute to the degenerative depletion of neurodegenerative processes, disruptions in cardiovascular homeostasis, and metabolic irregularities.^3^, ^4^, ^5^, ^6^ These interconnected effects influence every physiological organ system.

Nevertheless, it is essential to acknowledge that chronological age offers an imperfect approximation of the genuine biological aging state. The concept of biological age serves as a measure in assessing the state of bodily well-being, centering on the combined burden of pathophysiological alterations that influence mortality trends over the lifespan. A prominent illustration of this is phenotypic age (PhenoAge), an extensively employed gauge of biological aging, determined through the evaluation of nine multisystem clinical chemistry biomarkers.^7^ By accounting for multiple physiological factors, PhenoAge offers insights into the aging process that extends beyond traditional measures of time.

The shortening of telomere length also stands as a biological measure of human aging, offering insights into cellular aging processes and age-related disease susceptibility.^8^ Telomeres, the protective caps at the ends of chromosomes, have emerged as an area of study in the field of aging research.^9^ The gradual shortening of telomere length over time has garnered attention as a potential biological measure of human aging.^10^ This phenomenon, akin to the ticking of a cellular biological clock, reflects the interplay of genetics, environment, and lifestyle on the aging process. In vitro and in vivo studies suggest that telomere length might reflect the degree of cellular senescence and oxidative stress.^11^ However, from the epidemiology perspective, associations between telomere length with different biomarkers and diseases remains equivocal.^12^

Physical activity stands as a linchpin in the pursuit of active aging,^13^ offering a multitude of benefits for physical, mental, and social well-being.^14^, ^15^, ^16^ Habitual engagement in physical activity leads to improved aerobic fitness and enhanced muscle quality. These factors contribute to notable improvements in endocrine homeostasis, cardiovascular circulation, and brain cognitive function.^17^, ^18^, ^19^, ^20^ These cumulative body of evidence underscores the pivotal role of regular physical activity in preserving metabolic health and enhancing overall well-being across diverse populations. However, the relationship between physical activity and an extended life expectancy, though widely acknowledged, has been subject to limited investigation of its underlying mechanisms.

To the best of current knowledge, limited research, particularly large-scale population-based studies, have examined the interplay of physical activity, telomere length, and PhenoAge. In this cross-sectional research, our principal objectives are twofold: firstly, to delineate the relationships that link physical activity, telomere length, and PhenoAge, utilizing data from the National Health and Nutrition Examination Survey (NHANES); secondly, to quantify the extent of mediation exerted by telomere length in the pathway from physical activity to PhenoAge. Our hypothesis suggests a beneficial relationship between higher levels of physical activity, longer telomere length, and a correspondingly younger PhenoAge.

## 2 Materials and methods

### 2.1 Study population

The NHANES program, conducted by the U.S. Centers for Disease Control and Prevention (CDC), aims at monitoring the health and nutritional status of individuals across the United States. Imbued with a multi-level sampling strategy, the outcomes derived from this investigation possess significant nationwide generalizability. The data collection process unfolds over two-year cycles, ensuring a comprehensive and dynamic portrayal of the nation's health landscape. The program aforementioned had obtained ethical sanction from the NCHS Ethics Review Board. Participants involved in this research all provided written informed consent before engaging in the survey. This study followed the Strengthening the Reporting of Observational Studies in Epidemiology (STROBE) guidelines for cross-sectional studies.

Leukocyte telomere length—an indicator for understanding cellular aging—is calculated using DNA samples procured exclusively during the years 1999–2002, spanning a concise period of four years. The telomere data was made publicly accessible in November 2014. During the years 1999–2000 and 2001–2002, NHANES conducted a request for adult participants aged 20 years or older to provide DNA samples for analysis. Initially, a total of 18,759 participants were included in the study, out of which 7826 respondents aged 20 years and older had telomere data available. However, 55 of these participants did not complete the necessary tests to collect blood biomarker information, resulting in a final sample size of 7765 participants for calculating phenotypic age. Afterward, 1565 participants without covariate data were excluded from the analysis, leading to a final study population comprising 6200 participants. Due to the complex survey design of NHANES, which involves multistage stratified sampling and sample weighting, traditional power calculations (e.g., using G∗Power) are not directly applicable.

### 2.2 Measurement

#### 2.2.1 Physical activity measurement

The Global Physical Activity Questionnaire (GPAQ) was used to assess physical activity levels. Physical activity was measured using the responses of subjects about their participation in specific activities (such as walking, swimming, different ball games) during the past month. Subjects recorded the number of days in the past month and the usual number of minutes per bout they engaged in each activity. The intensity of their involvement as moderate or vigorous, based on definitions provided by NHANES.^21^ Each activity was assigned a predetermined metabolic equivalent of task (MET) value according to its reported intensity. To calculate the MET score for each activity, the compendium of physical activity was employed, with MET values assigned by NHANES for the 62 activities duly noted on the NHANES website.

From the frequency and duration information, total minutes per week of each participant was added to calculate the total minutes. The quantification of physical activity was conducted using MET-minutes, a method that represents the ratio between an individual's metabolic rate during physical exertion and at rest. Multiplying MET values with time and frequency, the total MET-minutes per week can be estimated for the residents' level of physical activity participation.^22^ While the GPAQ has been widely used in epidemiological studies, its criterion-related validity may vary depending on the population. The validity of the GPAQ in populations similar to the current study cohort, as well as the validity of self-reported MET-minutes in predicting health outcomes such as depression,^23^ cognitive function,^24^ and sleep disturbances,^25^ has been demonstrated in previous studies. Moreover, the criterion-related validity of the GPAQ has been evaluated in U.S. adults, demonstrating moderate to strong correlations with accelerometer-based measures in large-scale validation studies, supporting its use in population-based research.^26^^,^^27^

#### 2.2.2 Telomere length measurement

The NHANES study included a procedure for measuring telomere length in participants. To assess telomere length, DNA samples from participants were obtained and subjected to multiple laboratory techniques. Briefly, these techniques involved quantitative polymerase chain reaction (qPCR) assays specifically designed to measure telomere repeats relative to a single-copy gene. In the NHANES study of telomere length measurement conducted during the period of 1999–2002, analyses were undertaken to ensure data integrity and precision. Firstly, any potential outlier samples, constituting less than 2 % of the total samples, were identified and excluded from all subsequent calculations. This step aimed to maintain the robustness of the dataset by eliminating any aberrant values that might confound the overall analysis.

Subsequently, the Telomere-to-Single Copy Gene (T/S) ratio, a widely employed standard for estimating telomere length relative to standard reference DNA, was calculated using standard protocols. The T/S ratio reflects the relative abundance of telomeric DNA compared to the single-copy gene within each sample. To evaluate the variability of the assay measurements, the interassay coefficient of variation was determined to be 6.5 %. This strategy quantifies the degree of variation between repeated measurements in different assays, serving as an important quality control parameter. Further analyses involved the conversion of mean T/S ratio values to base pairs, enabling a more concrete representation of telomere length.^28^ This transformation was achieved by applying the formula: 3274 + 2413 × (T/S), yielding telomere length estimates expressed in base pairs.

#### 2.2.3 Phenotypic age measurement

Phenotypic age, as measured by the established PhenoAge algorithm, serves as a comprehensive indicator of an individual's biological aging status.^29^ In accordance with the formula developed by Levine et al., PhenoAge utilizes age and nine distinct biomarkers to generate an informative assessment.^7^ The calculation of PhenoAge involves the incorporation of several key biomarkers, namely albumin, creatinine, glucose, C-reactive protein, lymphocyte percentage, mean cell volume, red cell distribution width, alkaline phosphatase, and white blood cell count. These biomarkers were chosen based on their demonstrated relevance to physiological aging processes and their ability to provide meaningful insights into overall health. To derive the PhenoAge value for each participant in the study, relevant biomarker data was extracted from the NHANES Laboratory Data. Specifically, the following datasets were utilized: “Albumin & Creatinine—Urine, Plasma Fasting Glucose & Insulin, C-Reactive Protein (CRP), Complete Blood Count with 5-part Differential—Whole Blood, Standard Biochemistry Profile.” Through careful integration of these datasets and adherence to standardized protocols outlined in the Supplementary Methods, accurate and reliable PhenoAge calculations were achieved. The utilization of the PhenoAge algorithm, incorporating diverse biomarkers and precise computational methods, enables a comprehensive evaluation of an individual's biological age beyond chronological age alone.

#### 2.2.4 Covariate assessment

Drawing on established methodologies from previous studies, our analytical approach involved adjustments for the following covariates.^30^ Age, a pivotal demographic variable, was stratified into three distinct brackets: those aged less than 40, those aged 40 or older but less than 60, and those aged 60 or older. Furthermore, accounting for the influence of race, individuals were categorized into non-Hispanic White, non-Hispanic Black, Mexican American, or other race/ethnicity groups. Marital status, a psychosocial factor, was classified into three categories: never married, married or living with a partner, and widowed or divorced. Education level, a cornerstone of socio-economic status, was divided into three tiers: below high school, high school, and college or above.^31^ Economic considerations were encapsulated by the poverty income ratio, divided into three strata: less than 1, between 1 and 3, and 3 or greater. Body Mass Index (BMI), a strategy of physical health, saw participants categorized into underweight/healthy weight (BMI <25.0 kg/m^2^), overweight (BMI ≥25.0 and < 30.0 kg/m^2^), and obese (BMI ≥30.0 kg/m^2^) groups.^32^ Moreover, smoking status was categorized into three classifications: non-smokers, defined as those who had never smoked or had smoked fewer than 100 cigarettes in their lifetime; former smokers, characterized as those who had smoked at least 100 cigarettes but had discontinued the habit; and current smokers, encompassing participants who had smoked at least 100 cigarettes and reported a non-zero number of cigarettes per day in the past 30 days.^33^ Finally, patterns of alcohol consumption were examined, distinguishing between non-drinkers, moderate alcohol users, and high alcohol users. Moderate alcohol use was defined as 14 or fewer drinks per week for men, or 7 or fewer drinks per week for women, or 5 or fewer drinks per day on any single day within the past year for either gender. High alcohol use was defined as surpassing the threshold of 14 drinks per week for men, or 7 drinks per week for women, or consuming 5 or more drinks in a single day on at least one occasion within the past year for either men or women.^34^

### 2.3 Statistical analysis

The descriptive statistics of the study population were summarized using weighted means and standard errors for continuous variables, while categorical variables were expressed as weighted percentages. The normality of telomere length and PhenoAge was assessed using the Shapiro-Wilk test. Both variables showed right-skewed distributions, so logarithmic transformation was applied prior to regression analyses. Multivariate linear regression analyses were conducted to explore associations between PA, log-transformed telomere length, and log-transformed PhenoAge, with adjustments made for covariates. In accordance with standard practice in survey-weighted regression analyses, we reported β coefficients and 95 % confidence intervals to reflect population-level associations.

To probe deeper into the influence of covariates on these relationships, we implemented a three-part model approach: The Crude Model (unadjusted), Model 1 (adjusted for age, sex, and race/ethnicity), and Model 2 (fully adjusted model accounting for age, sex, race, marital status, education, poverty status, body mass index, smoking status, and alcohol consumption status). Subsequently, in addition to evaluating the direct effect of PA on PhenoAge, we embarked on an examination of the indirect (mediating) impact of telomere length on this association. This mediation analysis was performed to quantify the proportion of the relationship between PA and PhenoAge that is mediated by telomere length.

**Fig. 1:**
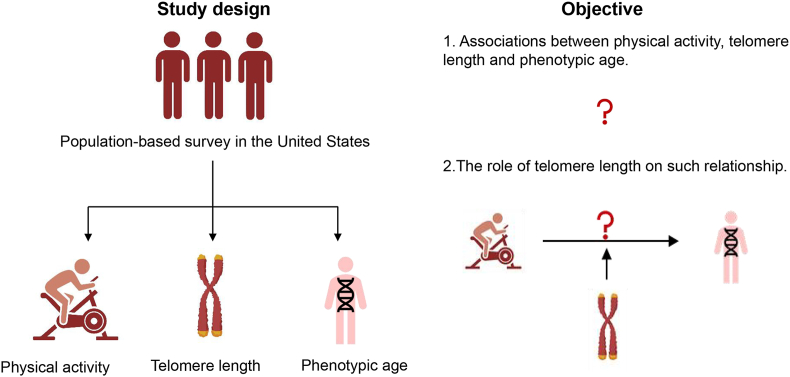
Study design and objective.

A schematic revealing the study design and objectives is presented in Fig. 1. All statistical analyses were executed using R version 4.2.0, bolstered by “mediation” and “survey” packages. Statistical significance was defined as a two-tailed p-value less than 0.05. Moreover, our analyses were conducted with consideration of the complex sampling weights in adherence to the guidelines by CDC, ensuring generalizability of study findings.

## 3 Results

Table 1 provides a summary of participant demographics stratified by their levels of physical activity. After applying inclusion and exclusion criteria, our final sample comprised 6200 individuals, representing a weighted population of 136,348,343, accounting for the weighted sampling methodology. Approximately half of the participants were male (49.17 %), with a mean telomere length of 5803.58 ± 33.57 base pairs and an average PhenoAge of 40.43 ± 0.42 years. The average PA participation in this study was 4891.65 MET-minutes/week. Notably, participants engaging in high levels of PA tended to be male, non-Hispanic White, and had educational attainments beyond college. Given that the participants constituted a large, national sample, relative activity levels (total MET-minutes) were used to form groups. PA groups were divided into quartiles: Sedentary (<40 MET-minutes/week), Low PA (≥40 and < 720 MET-minutes/week), Moderate PA (≥720 and < 4680 MET-minutes/week), and High PA (≥4680 MET-minutes/week). These cutoffs were derived from the quartile distribution of total MET-minutes/week in our study population.

**Table 1:** Demographic characteristics of study participants stratified by physical activity levels.

Variable	Total participants	Sedentary	Low PA	Moderate PA	High PA	P-value
Age						<0.001
<40	39.7	34.3	43.92	39.96	44.82	
[40, 60)	38.5	36.67	38.16	40.73	38.71	
≥ 60	21.8	29.03	17.93	19.3	16.46	
Sex						<0.001
Male	49.17	45.24	42.78	46.25	58.07	
Female	50.83	54.76	57.22	53.75	41.93	
Race/ethnicity						<0.001
Non-hispanic White	74.52	66.96	73.49	78.38	80.2	
Non-hispanic Black	8.56	12.44	7.79	6.31	6.21	
Mexican American	6.82	9.2	6.11	6.45	4.53	
Other Race/ethnicity	10.1	11.4	12.61	8.85	9.06	
Marital status						<0.001
Never married	15.58	13.32	11.95	14.54	20.09	
Married/living with partner	66.73	64.75	70.86	69.48	65.54	
Widowed/divorced	17.7	21.93	17.19	15.98	14.37	
Education						<0.001
Below high school	6.28	12.24	4.82	3.61	2.01	
High school	39.86	50.17	39.68	34.29	32.76	
College or above	53.87	37.59	55.5	62.09	65.23	
Poverty income ratio						<0.001
< 1	13.14	19.53	13.4	10.06	8.33	
[1,3)	35.7	44.23	38.76	31.9	28.3	
≥ 3	51.16	36.24	47.83	58.05	63.37	
BMI (kg/m^2^)						<0.001
< 25	34.48	31.13	31.63	34.74	38.9	
[25, 30)	34.8	32.29	33.28	35.45	37.57	
≥ 30	30.72	36.58	35.09	29.81	23.53	
Smokers						<0.001
Never smoker	49.73	45.29	48.47	51.82	53.4	
Former smoker	25.63	25.26	23.05	25.78	26.56	
Current smoker	24.64	29.45	28.48	22.4	20.04	
Alcohol drinkers						<0.001
Nondrinker	29.29	38.57	32.65	23.9	22.39	
Moderate alcohol use	49.67	40.8	46.13	55.77	55.47	
High alcohol use	21.04	20.63	21.21	20.33	22.14	
Telomere length (base pairs)	5803.58 ± 33.57	5737.86 ± 40.97	5797.09 ± 48.86	5801.47 ± 31.31	5884.98 ± 33.1	<0.001
Phenotypic age (years)	40.43 ± 0.42	45.1 ± 0.58	38.93 ± 0.96	38.75 ± 0.51	36.8 ± 0.75	<0.001

Notes: **∗**Weighted percentage for category variables and weighted Mean ± SE for continuous variables: NHANES, National Health and Nutrition Examination Survey; BMI, body mass index; PA, physical activity; For categorical variables: survey-weighted percentage, P-value was by survey-weighted Chi-square test; For continuous variables: survey-weighted mean ± SE, P-value was by survey-weighted linear regression.

Moving to Table 2, it demonstrates the relationship between PA levels and log-based PhenoAge. In the initial unadjusted model, employing the sedentary group as the reference, a statistically significant negative correlation was evident [Low PA, β (95 % CI): 0.142(−0.200,-0.084), p < 0.001; Moderate PA, β (95 % CI): 0.147(−0.183,-0.111), p < 0.001; High PA, β (95 % CI): 0.209(−0.262,-0.156), p < 0.001]. These findings maintained consistency in Model 1. In Model 2, following adjustments for a range of socio-demographic and health-related covariates, participants with higher levels of physical activity were associated with a lower PhenoAge [Low PA, β (95 % CI): 0.039(−0.071,-0.008), p = 0.021; Moderate PA, β (95 % CI): 0.058(−0.082,-0.034), p < 0.001; High PA, β (95 % CI): 0.069(−0.096,-0.042), p < 0.001]. Moreover, stratified results for associations between PA levels and log-based phenotypic age under different influencing factors were provided in Supplementary Table 1, which was consistent with our main findings.

**Table 2:** Weighted linear regression results for relationship between PA levels and log-based phenotypic age.

	Crude model^a^		Model 1^b^		Model 2^c^	
	β (95 % CI)	P-value	β (95 % CI)	P-value	β (95 % CI)	P-value
PA levels						
Sedentary	Reference		Reference		Reference	
Low PA	−0.142(-0.200,-0.084)	<0.001	−0.037(-0.068,-0.005)	0.025	−0.039(-0.071,-0.008)	0.021
Moderate PA	−0.147(-0.183,-0.111)	<0.001	−0.073(-0.098,-0.047)	<0.001	−0.058(-0.082,-0.034)	<0.001
High PA	−0.209(-0.262,-0.156)	<0.001	−0.099(-0.127,-0.070)	<0.001	−0.069(-0.096,-0.042)	<0.001

Notes.

a Crude model, no covariate was adjusted.

b Model 1, age, sex, and race were adjusted.

c Model 2, age, sex, race, marital status, education, poverty status, body mass index, smokers, alcohol drinkers were adjusted. CI, confidence interval. PA, physical activity.

Table 3 shows the relationship between PA levels and log-based telomere length. Using the sedentary group as the reference, the unadjusted model revealed a notable association between higher PA levels and extended telomere length [Low PA, β (95 % CI): 0.011(0.000,0.022), p = 0.046; Moderate PA, β (95 % CI): 0.012(0.002,0.022), p = 0.026; High PA, β (95 % CI): 0.026(0.016,0.035), p < 0.001]. Furthermore, in the fully adjusted Model 2, high PA demonstrated a positive correlation with telomere length [β (95 % CI): 0.011(0.001, 0.022), p = 0.034]. However, no statistically significant associations were observed in the low and moderate PA groups [Low PA, β (95 % CI): 0.001(−0.011, 0.012), p = 0.939; Moderate PA, β (95 % CI): 0.002(−0.007, 0.011), p = 0.646]. We also conducted a stratified analysis of the different covariates on the association between PA levels and telomere length in Supplementary Table 2 to verify the main results.

**Table 3:** Weighted linear regression results for relationship between PA levels and log-based telomere lengths.

	Crude model^a^		Model 1^b^		Model 2^c^	
	β (95 % CI)	P-value	β (95 % CI)	P-value	β (95 % CI)	P-value
PA levels						
Sedentary	Reference		Reference		Reference	
Low PA	0.011(0.000,0.022)	0.046	0.001(-0.010, 0.012)	0.816	0.001(-0.011, 0.012)	0.939
Moderate PA	0.012(0.002,0.022)	0.026	0.005(-0.005, 0.015)	0.284	0.002(-0.007, 0.011)	0.646
High PA	0.026(0.016,0.035)	<0.001	0.016(0.006, 0.027)	0.003	0.011(0.001, 0.022)	0.034

Notes.

a Crude model, no covariate was adjusted.

b Model 1, age, sex, and race were adjusted.

c Model 2, age, sex, race, marital status, education, poverty status, body mass index, smokers, alcohol drinkers were adjusted. CI, confidence interval. PA, physical activity.

**Fig. 2:**
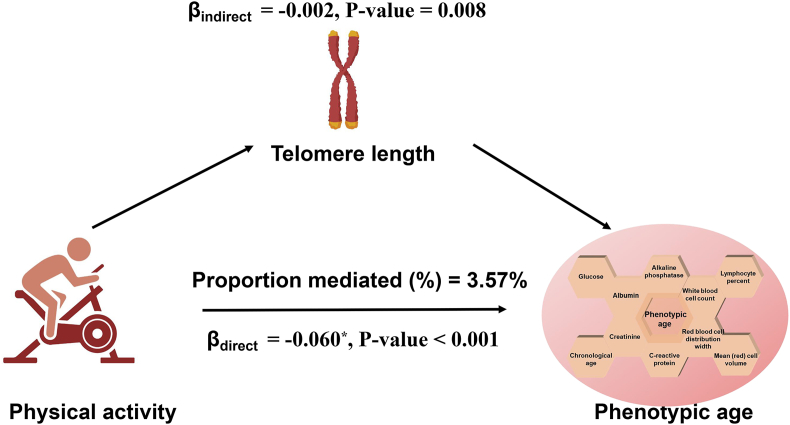
Path diagram of the mediation analysis.

Additionally, we conducted mediation analyses, the results of which are presented in Fig. 2. This involved PA participation as the exposure variable and log-transformed telomere length as the mediator. To examine these relationships, we employed Model 2, a fully adjusted model, for the mediation analysis. The findings indicated that log-transformed telomere length exerted significant mediating effects on the association between PA and log-based PhenoAge, accounting for approximately 3.57 % of the total mediation. Specifically, the direct effect on PhenoAge was −0.060 (p < 0.001), while the indirect effect via telomere length was −0.002 (p = 0.008).

## 4 Discussions

The present investigation, encompassing 6200 participants from the nationally representative NHANES cohort, showed a relationship between heightened levels of PA and a decelerated pace of biological aging, as indicated by PhenoAge. Furthermore, we investigated an additional aspect of the aging phenomenon, termed telomere length. Encouragingly, our findings highlighted a positive correlation between elevated PA levels and extended telomere length. Through mediation analysis, we demonstrated that the beneficial impact of PA on longevity may be, in part, explained by its role in slowing the process of telomere attrition. This insight provided a contribution to our understanding of the interplay between PA participation, biological aging, and longevity.

In recent years, research on aging has witnessed a paradigm shift from focusing solely on chronological age to considering biological markers that more accurately reflect the aging process. One such marker is PhenoAge, which encompasses a multi-dimensional assessment of age-related physiological changes.^35^^,^^36^ The beneficial effects of physical activity on phenotypic age can be elucidated through various physiological and psychological functions.^37^^,^^38^ An extensive prospective cohort study in the United States, encompassing over 10,000 middle-aged adults, revealed that sustaining activity levels during later stages of life were correlated with a reduced likelihood of heart failure development.^39^ These favorable adaptations contributed to a more youthful phenotype, characterized by enhanced organ function, reduced risk of chronic diseases, and improved overall well-being.^40^^,^^41^

Concurrently, telomere attrition, the gradual shortening of protective DNA sequences at the end of chromosomes, has emerged as a fundamental component of cellular aging.^42^^,^^43^ Emerging research has spotlighted PA as a modifiable lifestyle factor that exerts a notable influence on telomere dynamics, however, the evidence is controversial.^44^, ^45^, ^46^ One systematic review conducted a meta-analysis incorporating data from 11 studies found that the evidence implicating physical activity in telomere length (TL) regulation appears tenuous.^47^ This uncertainty mainly stemmed from several differences in the methods used across the analyzed studies. These discrepancies encompassed a risk of publication bias, dissimilarities in study populations, variations in the assessment of both PA levels and telomere length, as well as an inadequacy in accounting for confounding variables, such as comorbidities and other pertinent lifestyle factors.

Nevertheless, by using a nationwide sample and adjusting for multiple covariates, our study still supported the benefits of regular exercise in slowing down telomere shortening. Mechanistically, exercise-induced telomere preservation may be attributed to the activation of telomerase, an enzyme that counteracts telomere shortening, as well as the reduction of oxidative stress, inflammation and skeletal muscle satellite cells, which are known accelerators of telomere erosion.^48^^,^^49^ While telomere length is often associated with shortening over time, it is important to note that telomere regulation is a dynamic process, and physical activity may also play a role in the restoration or maintenance of telomere length.^50^^,^^51^ Regular physical activity has been shown to lower levels of C-reactive protein^52^ and mitigate systemic inflammation,^53^ both of which are critical factors in cellular aging and telomere erosion.

Our study suggested that the influence of physical activity on phenotypic age and longevity may, in part, be mediated by its effect on telomere shortening. By preserving telomere length, regular exercise potentially mitigated cellular aging and extended the replicative lifespan of cells.^54^^,^^55^ For example, similar to how PhenoAge mediates the effect of lifestyle factors on mortality, telomere length also mediates the relationship between physical activity and biological aging. A study on Life's Essential 8 (LE8) showed that PhenoAge mediated 36 % and 22 % of the effects of LE8 on all-cause and cardiovascular mortality, respectively.^56^ Specifically, PhenoAge mediated 30 %, 11 %, 9 %, and 7 % of the effects of diet, smoking, blood pressure, and physical activity, respectively, on all-cause mortality risk. These findings highlight the importance of multiple health factors, such as diet, inflammation, and oxidative stress, in the aging process. While our study emphasizes telomere length, further research is needed to fully understand the relative importance of these factors in how physical activity influences aging and longevity. This mediation role of telomeres underscores the significance of lifestyle interventions in the aging process.

This study demonstrated several strengths. Leveraging a community-based cohort in the United States, it contributed to the aging literature. Notably, it elucidated the protective association between PA and broader spectrum of biological aging, particularly emphasizing telomere-related processes. The inclusion of 6200 participants from varied socio-demographic backgrounds strengthens the external validity of the findings, allowing for broader applicability to different demographic groups. Last but not least, the study's methodological rigor is evident in its adjustment for a range of covariates, including age, sex, and socio-economic status, to control for potential confounding variables.

However, there are some limitations that should be noted. The primary constraint lay in the cross-sectional design, wherein the assessments of PA, telomere length, and PhenoAge were concurrent with participant recruitment. This temporal alignment restricted our capacity to infer causality in the observed associations. Additionally, the calculation of alternative aging metrics, like the DNAm PhenoAge, valued for its potential in gauging risks across various aging outcomes,^57^^,^^58^ was precluded due to the absence of pertinent DNA methylation data. Moreover, PA data were collected over a single month by questionnaires, which may not fully capture lifetime fluctuations in activity levels. Future research using longitudinal data would better address how long-term activity patterns influence telomere length and biological aging.

## 5 Conclusion

This study presented a comprehensive analysis of the relationship between PA, telomere length, and PhenoAge in a cohort of 6200 participants sourced from the NHANES dataset. We gained a better understanding of how PA correlated with aging at both the cellular level (telomere length), as well as the phenotypic level (PhenoAge). Additionally, the results offered evidence of PA's association with delay of telomere attrition, adding the dimension to our knowledge of healthy aging. Notably, mediation analysis revealed that telomere length played a significant role in mediating the association between PA and PhenoAge, further elucidating the mechanisms underlying the aging process. These findings emphasized the role of PA in shaping the biological markers of aging and provided valuable contributions to the growing field of aging research, advocating for further biological studies and randomized controlled trials to confirm and expand upon our findings.

## CRediT authorship contribution statement

**Yanwei You:** Conceptualization, Methodology, Formal analysis, Validation, Investigation, Writing – original draft, Writing – review & editing. **Dizhi Wang:** Writing – original draft, Visualization, Validation, Writing – review & editing. **Hao Ding:** Writing – original draft, Visualization, Validation, Writing – review & editing. **Weizhao Wang:** Methodology, Investigation. **Qiyu Liu:** Methodology, Investigation. **Danyi Zhang:** Validation, Writing – review & editing. **Yuquan Chen:** Writing – review & editing, Supervision, Project administration. **Xindong Ma:** Conceptualization, Supervision, Project administration, Funding acquisition.

## Ethics approval and consent to participate

The National Health and Nutrition Examination Survey (NHANES) is a publicly accessible database approved by the Institutional Review Board (IRB) of the National Center for Health Statistics. All participants provided written informed consent during their participation in the national survey. As secondary analysis does not require additional IRB approval, this study was exempt from further ethical review and approval.

## Disclosure statement

The authors have nothing to disclose.

## Data availability

Data will be made available on request.

## Funding

This study was supported by the 10.13039/501100004147Institute of Sports Development Research of Tsinghua University (Research on John Mo's thought and practice of Physical Education); Tsinghua University Commissioned Project by Enterprises and Public Institutions (China National Children's Center) – 20232000264; Tsinghua University Initiative Scientific Research Program (2024THZWYY05). Tsinghua University Computational Social Science Research Program (202501020008).

## Declaration of competing interest

The authors declare that they have no known competing financial interests or personal relationships that could have appeared to influence the work reported in this paper.
