# Supplementary material for: Mediation role of telomere length in the relationship between physical activity and PhenoAge: A population-based study
Source: J Exerc Sci Fit. 2025 Mar 26;23(3):149–56. doi: 10.1016/j.jesf.2025.03.004 (PMC11994304; doi:10.1016/j.jesf.2025.03.004)
Supplement: Multimedia component 1 [file mmc1.docx]

Supplementary methods for calculating phenotypic age.

The calculation method for phenotypic age was as follows:


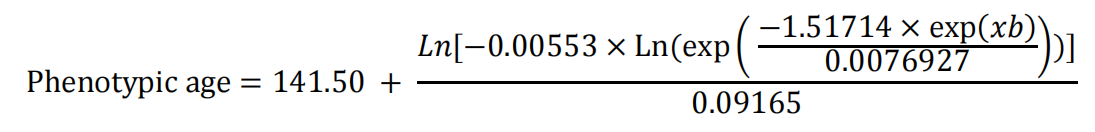


In the formula, the parameter xb = −19.907 + 0.0804 × chronological age − 0.0336 × albumin + 0.0095 × creatinine + 0.1953 × glucose + 0.0954 × Ln (C-reactive protein) − 0.0120 × lymphocyte percent + 0.0268 × mean cell volume + 0.3306 × red cell distribution width + 0.00188 × alkaline phosphatase + 0.0554 × white blood cell count.

Table S1. Weighted linear regression of stratified results for associations between PA levels and log-based phenotypic age.

|  | Sedentary | Low PA | P-value | Moderate PA | P-value | High PA | P-value | P for trend |
| --- | --- | --- | --- | --- | --- | --- | --- | --- |
| Age |  |  |  |  |  |  |  |  |
| < 40 | Reference | -0.047(-0.112, 0.018) | 0.152 | -0.064(-0.112,-0.016) | 0.011 | -0.126(-0.188,-0.065) | <0.001 | <0.001 |
| [40, 60) | Reference | -0.041(-0.084, 0.001) | 0.055 | -0.085(-0.126,-0.044) | <0.001 | -0.065(-0.105,-0.024) | 0.003 | <0.001 |
| ≥ 60 | Reference | -0.043(-0.088, 0.001) | 0.057 | -0.052(-0.074,-0.031) | <0.001 | -0.07(-0.093,-0.047) | <0.001 | <0.001 |
| Sex |  |  |  |  |  |  |  |  |
| Male | Reference | -0.082(-0.147,-0.017) | 0.015 | -0.091(-0.140,-0.041) | <0.001 | -0.175(-0.242,-0.108) | <0.001 | <0.001 |
| Female | Reference | -0.189(-0.257,-0.121) | <0.001 | -0.193(-0.249,-0.137) | <0.001 | -0.247(-0.323,-0.171) | <0.001 | <0.001 |
| Race/ethnicity |  |  |  |  |  |  |  |  |
| Non-hispanic White | Reference | -0.168(-0.229,-0.107) | <0.001 | -0.177(-0.224,-0.131) | <0.001 | -0.239(-0.299,-0.179) | <0.001 | <0001 |
| Non-hispanic Black | Reference | -0.168(-0.344, 0.007) | 0.059 | -0.175(-0.273,-0.076) | 0.001 | -0.293(-0.361,-0.225) | <0.0001 | <0.001 |
| Mexican American | Reference | -0.174(-0.308,-0.041) | 0.013 | -0.025(-0.095, 0.044) | 0.455 | -0.137(-0.209,-0.064) | <0.001 | 0.003 |
| Other race/ethnicity | Reference | -0.085(-0.255, 0.084) | 0.308 | -0.159(-0.288,-0.029) | 0.019 | -0.231(-0.353,-0.110) | <0.001 | <0.001 |
| Marital status |  |  |  |  |  |  |  |  |
| Never married | Reference | -0.097(-0.308, 0.113) | 0.351 | -0.121(-0.216,-0.026) | 0.014 | -0.264(-0.368,-0.159) | <0.0001 | <0.001 |
| Married/living with partner | Reference | -0.126(-0.193,-0.059) | <0.001 | -0.115(-0.165,-0.065) | <0.001 | -0.111(-0.163,-0.058) | <0.001 | <0.001 |
| Widowed/ divorced | Reference | -0.194(-0.282,-0.106) | <0.001 | -0.179(-0.242,-0.115) | <0.001 | -0.237(-0.308,-0.167) | <0.0001 | <0.001 |
| Poverty income ratio |  |  |  |  |  |  |  |  |
| < 1 | Reference | -0.148(-0.292,-0.004) | 0.045 | -0.313(-0.446,-0.179) | <0.001 | -0.44(-0.600,-0.280) | <0.0001 | <0.001 |
| [1,3) | Reference | -0.218(-0.301,-0.134) | <0.001 | -0.181(-0.262,-0.100) | <0.001 | -0.266(-0.339,-0.194) | <0.0001 | <0.001 |
| ≥ 3 | Reference | -0.084(-0.160,-0.008) | 0.032 | -0.106(-0.162,-0.049) | <0.001 | -0.16(-0.212,-0.108) | <0.0001 | <0.001 |
| Education |  |  |  |  |  |  |  |  |
| Below high school | Reference | -0.03(-0.265,0.206) | 0.796 | -0.01(-0.107,0.086) | 0.825 | -0.179(-0.433,0.074) | 0.158 | 0.155 |
| High school | Reference | -0.176(-0.263,-0.089) | <0.001 | -0.146(-0.213,-0.078) | <0.001 | -0.222(-0.310,-0.135) | <0.001 | <0.001 |
| College or above | Reference | -0.096(-0.177,-0.016) | 0.021 | -0.118(-0.168,-0.067) | <0.001 | -0.164(-0.221,-0.107) | <0.001 | <0.001 |
| BMI (kg/m^2^) |  |  |  |  |  |  |  |  |
| < 25 | Reference | -0.285(-0.403,-0.168) | <0.001 | -0.181(-0.266,-0.096) | <0.001 | -0.258(-0.356,-0.161) | <0.0001 | <0.001 |
| [25, 30) | Reference | -0.069(-0.152, 0.015) | 0.102 | -0.134(-0.194,-0.075) | <0.001 | -0.15(-0.219,-0.082) | <0.001 | <0.001 |
| ≥ 30 | Reference | -0.074(-0.152, 0.005) | 0.065 | -0.098(-0.142,-0.054) | <0.001 | -0.168(-0.246,-0.091) | <0.001 | <0.001 |
| Smokers |  |  |  |  |  |  |  |  |
| Never smoker | Reference | -0.123(-0.220,-0.026) | 0.015 | -0.137(-0.190,-0.085) | <0.001 | -0.248(-0.325,-0.171) | <0.001 | <0.001 |
| Former smoker | Reference | -0.127(-0.221,-0.032) | 0.011 | -0.108(-0.178,-0.038) | 0.004 | -0.117(-0.183,-0.051) | 0.001 | 0.001 |
| Current smoker | Reference | -0.18(-0.277,-0.083) | <0.001 | -0.224(-0.292,-0.155) | <0.001 | -0.253(-0.332,-0.175) | <0.001 | <0.001 |
| Alcohol drinkers |  |  |  |  |  |  |  |  |
| Nondrinker | Reference | -0.176(-0.245,-0.106) | <0.001 | -0.167(-0.241,-0.094) | <0.001 | -0.264(-0.414,-0.114) | 0.001 | <0.001 |
| Moderate alcohol use | Reference | -0.115(-0.202,-0.028) | 0.012 | -0.082(-0.125,-0.039) | <0.001 | -0.121(-0.165,-0.078) | <0.001 | <0.001 |
| High alcohol use | Reference | -0.107(-0.202,-0.012) | 0.029 | -0.186(-0.263,-0.110) | <0.001 | -0.248(-0.339,-0.157) | <0.001 | <0.001 |

Notes: Sedentary was used as the reference. Abbreviations: BMI, body mass index; PA, physical activity.

Table S2. Weighted linear regression of stratified results for associations between PA levels and log-based telomere lengths.

|  | Sedentary | Low PA | P-value | Moderate PA | P-value | High PA | P-value | P for trend |
| --- | --- | --- | --- | --- | --- | --- | --- | --- |
| Age |  |  |  |  |  |  |  |  |
| < 40 | Reference | -0.016(-0.032,0.001) | 0.058 | -0.005(-0.020,0.011) | 0.535 | 0.012(-0.001,0.025) | 0.075 | 0.09 |
| [40, 60) | Reference | 0.012(-0.012,0.036) | 0.327 | 0.013(-0.004,0.030) | 0.132 | 0.015( 0.001,0.028) | 0.032 | 0.035 |
| ≥ 60 | Reference | 0.023(-0.001,0.046) | 0.056 | -0.002(-0.019,0.014) | 0.758 | 0.02( 0.003,0.038) | 0.026 | 0.12 |
| Sex |  |  |  |  |  |  |  |  |
| Male | Reference | -0.003(-0.018,0.012) | 0.689 | 0.004(-0.010,0.018) | 0.545 | 0.018( 0.004,0.032) | 0.012 | 0.014 |
| Female | Reference | 0.023(0.006,0.040) | 0.008 | 0.017(0.002,0.031) | 0.026 | 0.037(0.022,0.051) | <0.0001 | <0.001 |
| Race/ethnicity |  |  |  |  |  |  |  |  |
| Non-hispanic White | Reference | 0.011(-0.001,0.023) | 0.077 | 0.018( 0.008,0.028) | 0.001 | 0.034( 0.022,0.045) | <0.001 | <0.001 |
| Non-hispanic Black | Reference | 0.003(-0.037,0.044) | 0.871 | 0.012(-0.009,0.033) | 0.257 | 0.021( 0.006,0.037) | 0.009 | 0.022 |
| Mexican American | Reference | 0.02(-0.004,0.045) | 0.097 | 0.01(-0.011,0.031) | 0.327 | 0.005(-0.019,0.028) | 0.674 | 0.542 |
| Other race/ethnicity | Reference | 0.037( 0.008,0.065) | 0.014 | -0.011(-0.039,0.017) | 0.422 | 0.016(-0.019,0.051) | 0.351 | 0.615 |
| Marital status |  |  |  |  |  |  |  |  |
| Never married | Reference | 0.002(-0.028,0.032) | 0.891 | 0.026( 0.002,0.051) | 0.036 | 0.02(-0.004,0.044) | 0.099 | 0.06 |
| Married/living with partner | Reference | 0.011(-0.002,0.023) | 0.087 | 0.006(-0.006,0.019) | 0.309 | 0.018( 0.006,0.030) | 0.006 | 0.015 |
| Widowed/ divorced | Reference | 0.02(-0.006,0.046) | 0.132 | 0.005(-0.015,0.026) | 0.605 | 0.04( 0.021,0.058) | <0.001 | 0.001 |
| Poverty income ratio |  |  |  |  |  |  |  |  |
| < 1 | Reference | 0.019(-0.010,0.049) | 0.194 | 0.03(-0.001,0.061) | 0.055 | 0.048( 0.005,0.091) | 0.031 | 0.022 |
| [1,3) | Reference | 0.008(-0.013,0.028) | 0.446 | 0.014( 0.001,0.027) | 0.041 | 0.027( 0.014,0.039) | <0.001 | <0.001 |
| ≥ 3 | Reference | 0.014(-0.001,0.028) | 0.069 | 0.007(-0.009,0.022) | 0.400 | 0.024( 0.009,0.039) | 0.003 | 0.007 |
| Education |  |  |  |  |  |  |  |  |
| Below high school | Reference | 0(-0.039,0.040) | 0.992 | 0(-0.030,0.030) | 0.993 | 0.022(-0.012,0.055) | 0.190 | 0.395 |
| High school | Reference | 0.004(-0.015,0.022) | 0.678 | 0.002(-0.014,0.017) | 0.818 | 0.014( 0.003,0.026) | 0.017 | 0.059 |
| College or above | Reference | 0.014( 0.000,0.028) | 0.053 | 0.011(-0.001,0.024) | 0.069 | 0.027( 0.012,0.041) | <0.001 | 0.001 |
| BMI (kg/m^2^) |  |  |  |  |  |  |  |  |
| < 25 | Reference | 0.02(-0.001,0.041) | 0.064 | 0.025( 0.007,0.043) | 0.009 | 0.039( 0.023,0.055) | <0.0001 | <0.001 |
| [25, 30) | Reference | 0.022( 0.002,0.042) | 0.032 | 0.009(-0.003,0.021) | 0.120 | 0.025( 0.009,0.041) | 0.004 | 0.006 |
| ≥ 30 | Reference | -0.005(-0.029,0.019) | 0.691 | -0.002(-0.020,0.015) | 0.782 | 0.009(-0.002,0.020) | 0.116 | 0.299 |
| Smokers |  |  |  |  |  |  |  |  |
| Never smoker | Reference | 0.018(0.007,0.029) | 0.003 | 0.014(0.003,0.026) | 0.014 | 0.034(0.020,0.048) | <0.001 | <0.001 |
| Former smoker | Reference | -0.001(-0.030,0.029) | 0.966 | 0.006(-0.013,0.025) | 0.492 | 0.022( 0.006,0.038) | 0.009 | 0.015 |
| Current smoker | Reference | 0.012(-0.016,0.040) | 0.393 | 0.012(-0.009,0.033) | 0.242 | 0.019(-0.004,0.042) | 0.106 | 0.115 |
| Alcohol drinkers |  |  |  |  |  |  |  |  |
| Nondrinker | Reference | 0.022(0.003,0.040) | 0.023 | 0.021(0.004,0.037) | 0.017 | 0.038(0.011,0.066) | 0.008 | 0.007 |
| Moderate alcohol use | Reference | 0.005(-0.012,0.022) | 0.529 | 0.003(-0.010,0.016) | 0.595 | 0.022( 0.010,0.035) | 0.001 | 0.003 |
| High alcohol use | Reference | 0.005(-0.018,0.028) | 0.660 | 0.008(-0.012,0.027) | 0.420 | 0.01(-0.011,0.030) | 0.350 | 0.331 |

Notes: Sedentary was used as the reference. Abbreviations: BMI, body mass index; PA, physical activity.
